# Supplementary material for: Multiple lineages of Streptomyces produce antimicrobials within passalid beetle galleries across eastern North America
Source: eLife. 2021 May 4;10:e65091. doi: 10.7554/eLife.65091 (PMC8096431; doi:10.7554/eLife.65091)
Supplement: Supplementary file 2. [file elife-65091-supp2.pdf]

(1) Actinomycin D

$[M+H]^+$

Mass error: 1.7 ppm

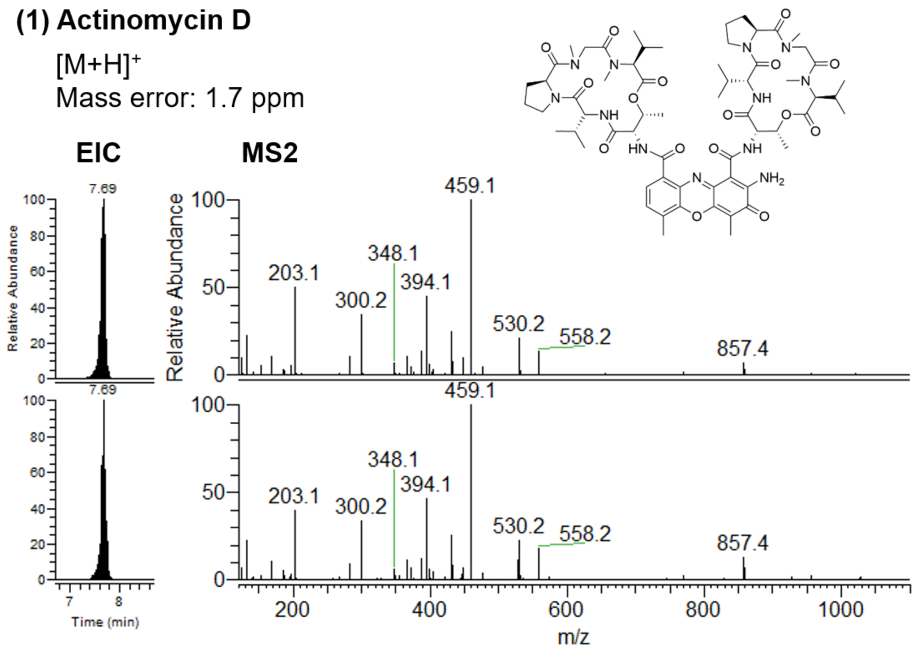

Standard

NL: 1.36E6

021620_Std_ActD#3128-3223 RT: 7.63-7.72 AV: 2 F: FTMS + p ESI d Full ms2 1255.6366@hcd30.00 [50.0000-1300.0000]

Strain P333

NL: 6.63E6

011319_EtAcMS2_P333#3913-4025 RT: 7.67-7.77 AV: 2 F: FTMS + p ESI d Full ms2 1255.6366@hcd30.00 [50.0000-1300.0000]

(2) Actinomycin X2

$[M+H]^+$

Mass error: 2.2 ppm

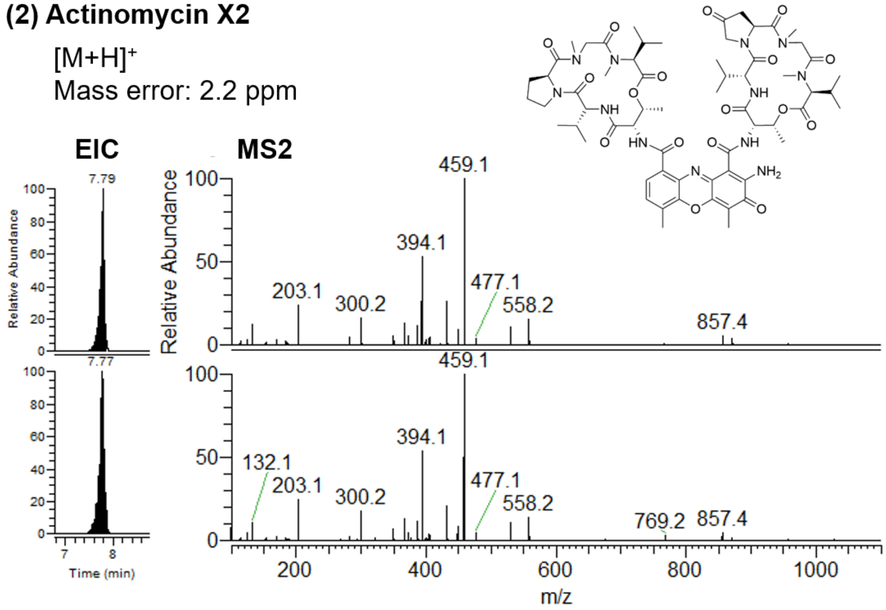

Standard

NL: 1.61E6

052120_Std_ActinomycinX2#3188-3253 RT: 7.74-7.82 AV: 2 F: FTMS + p ESI d Full ms2 1269.6119@hcd30.00 [50.0000-1315.0000]

Strain P333

NL: 1.90E7

011319_EtAcMS2_P333#3969-4052 RT: 7.74-7.84 AV: 2 F: FTMS + p ESI d Full ms2 1269.6119@hcd30.00 [50.0000-1315.0000]

(3) STA-21

$[M+H]^+$

Mass error: 1.6 ppm

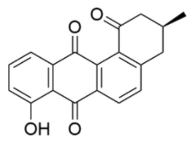

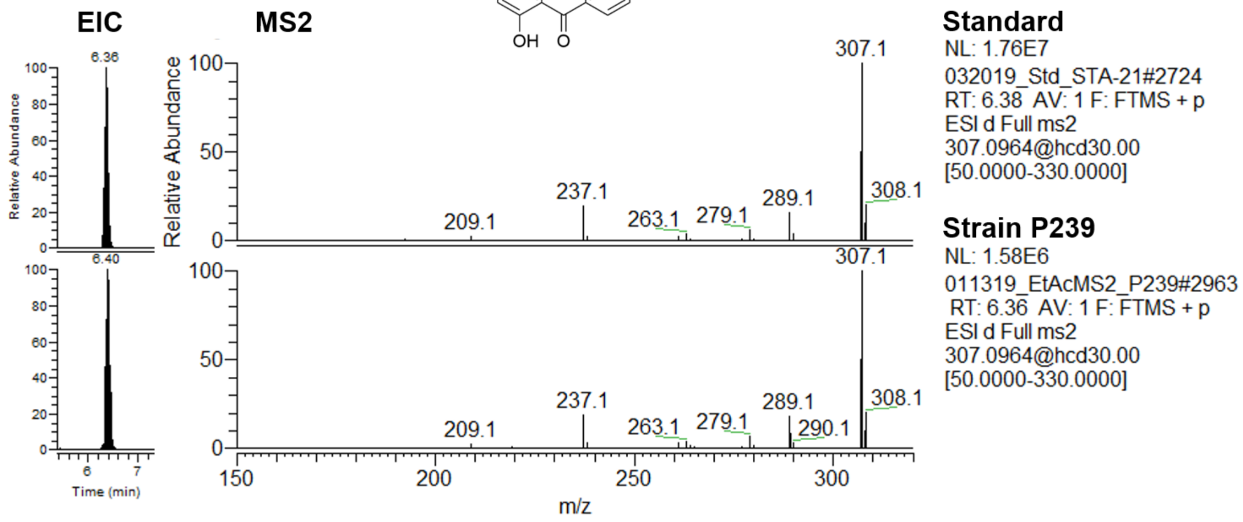

(4) Rubiginone B2

$[M+H]^+$

Mass error: 2.2 ppm

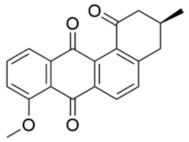

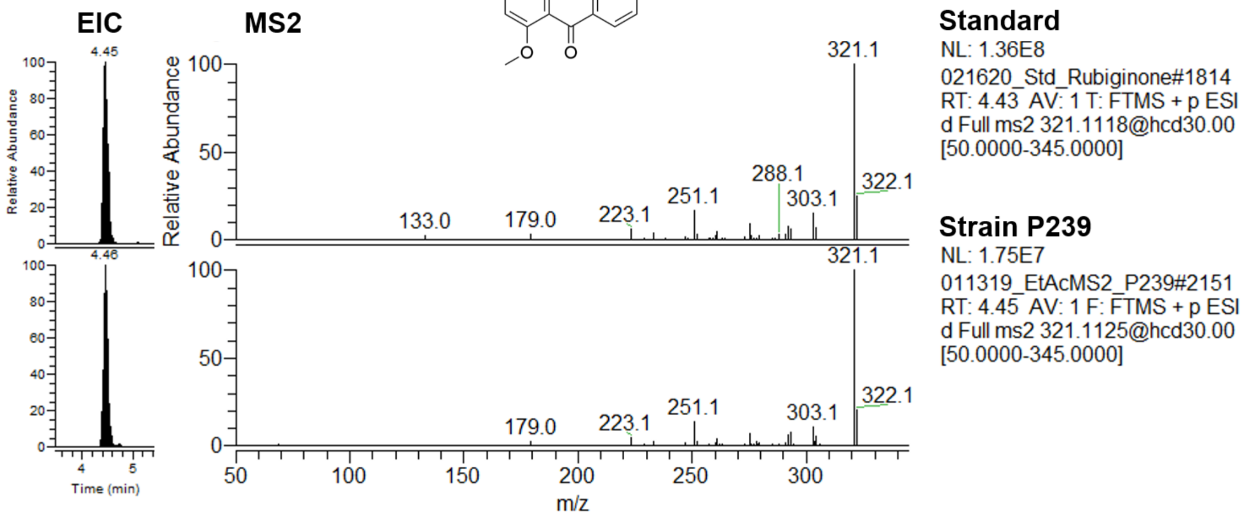

(5) Cycloheximide

$[M+H]^+$

Mass error: 2.1 ppm

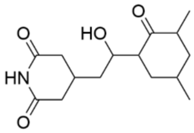

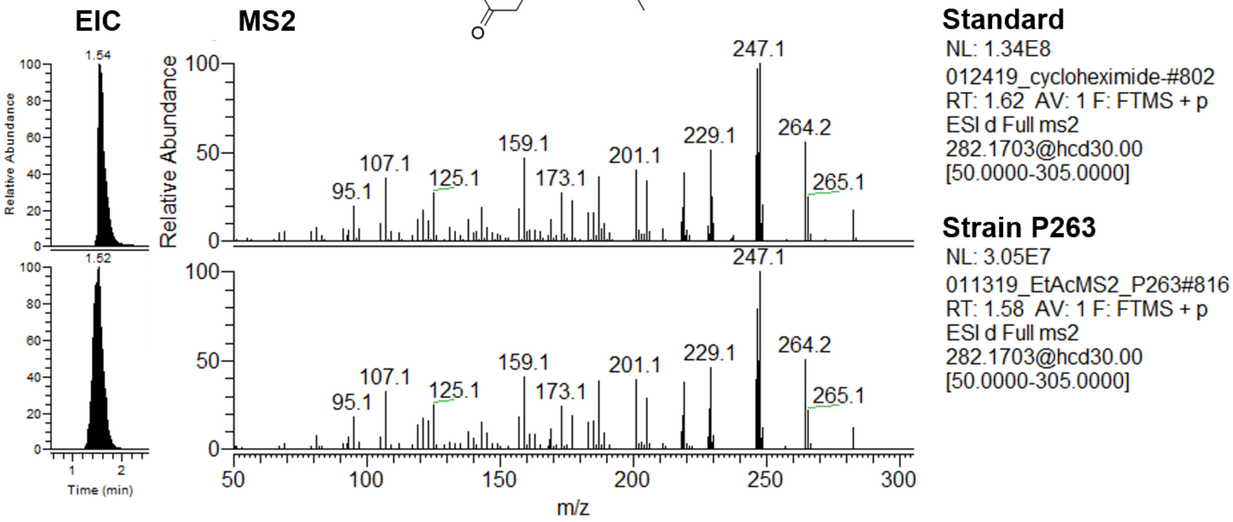

(6) Nonactin

$[M+Na]^+$

Mass error: 2.6 ppm

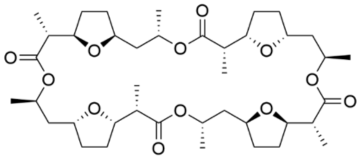

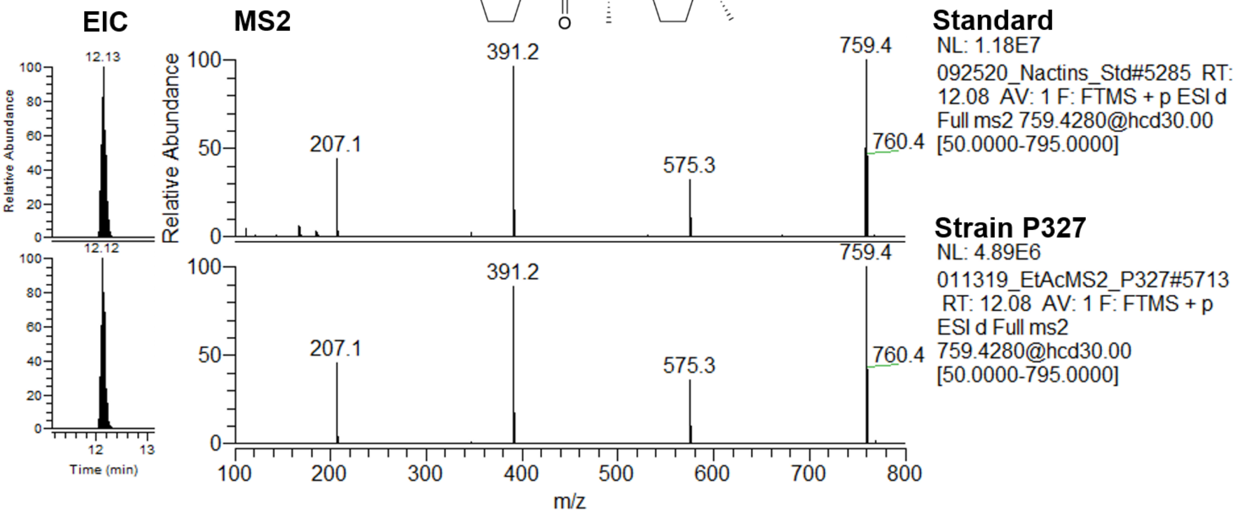

(7) Monactin

$[M+Na]^+$

Mass error: 3.2 ppm

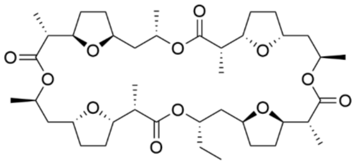

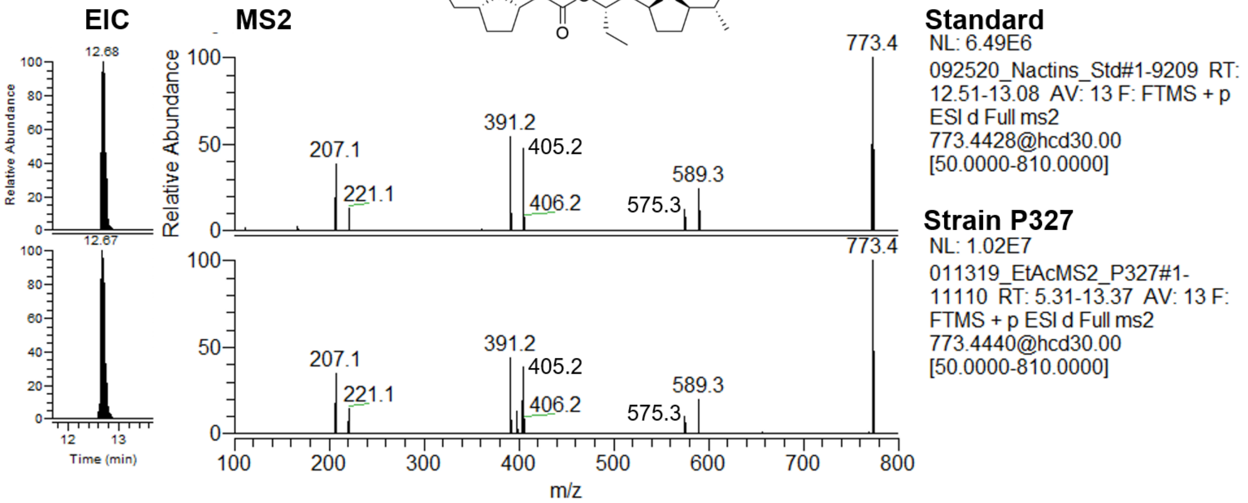

(8) Dinactin

$[M+Na]^+$

Mass error: 3.4 ppm

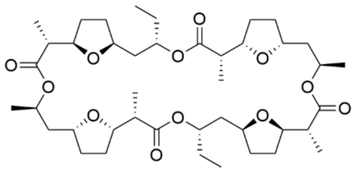

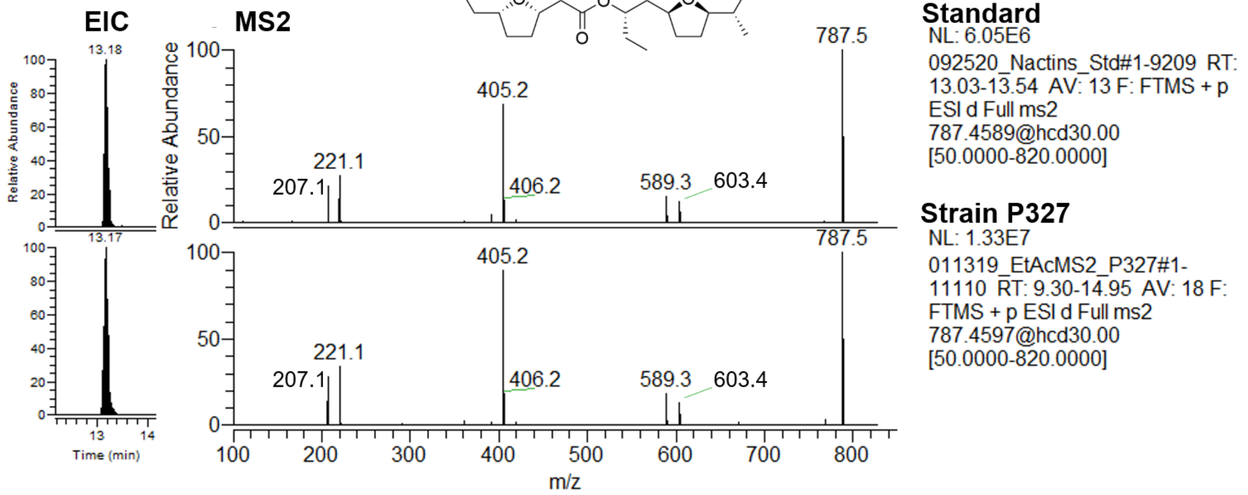

(9) Trinactin

[M+Na]+
Mass error: 2.5 ppm

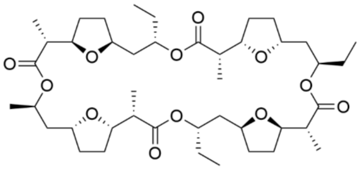

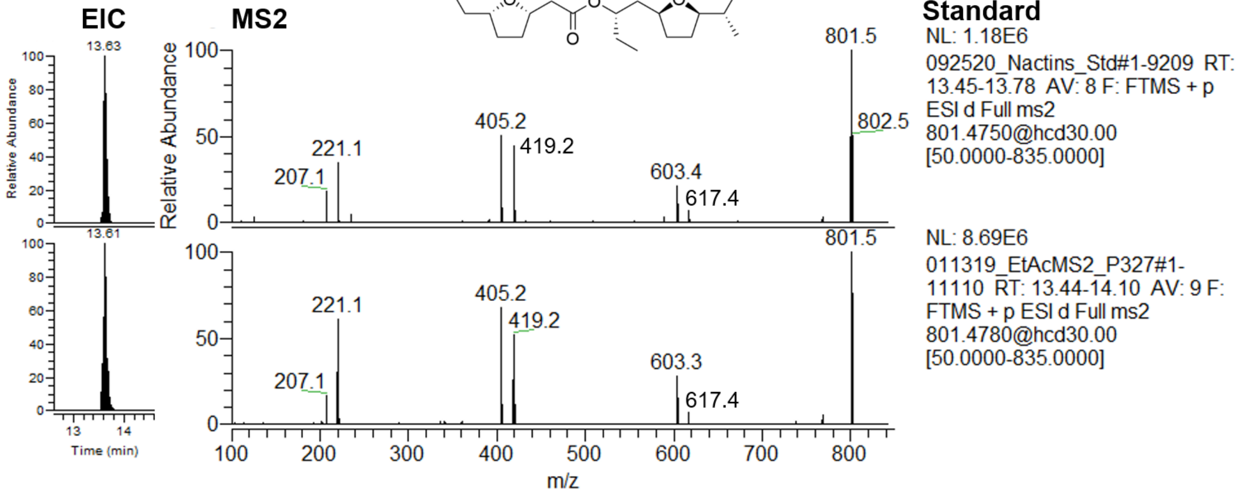

(10) Tetranactin

[M+Na]+
Mass error: 1.7 ppm

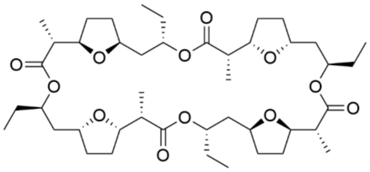

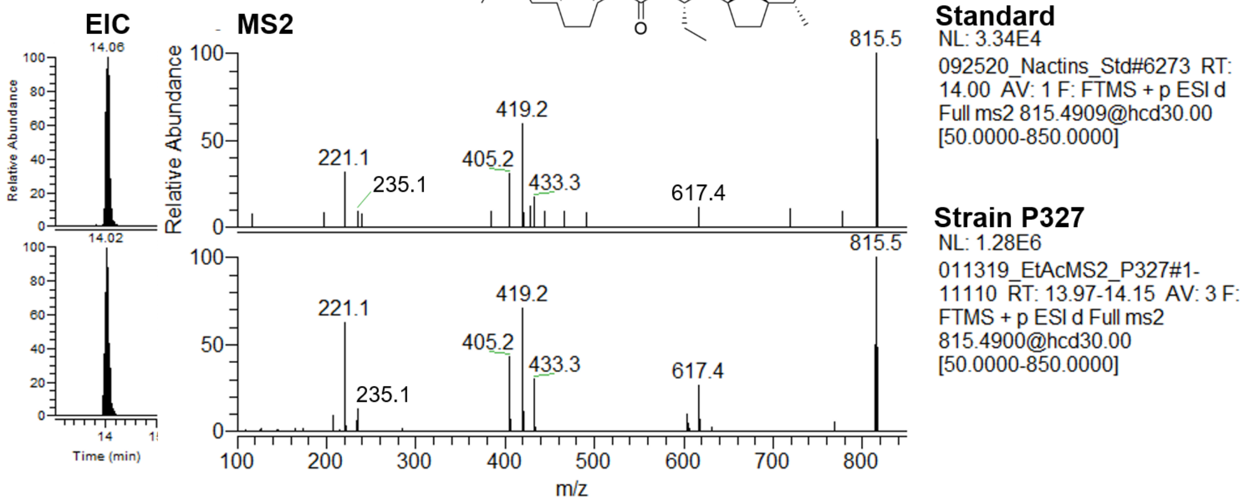

(11) Filipin I

$[M+Na]^+$

Mass error: 1.1 ppm

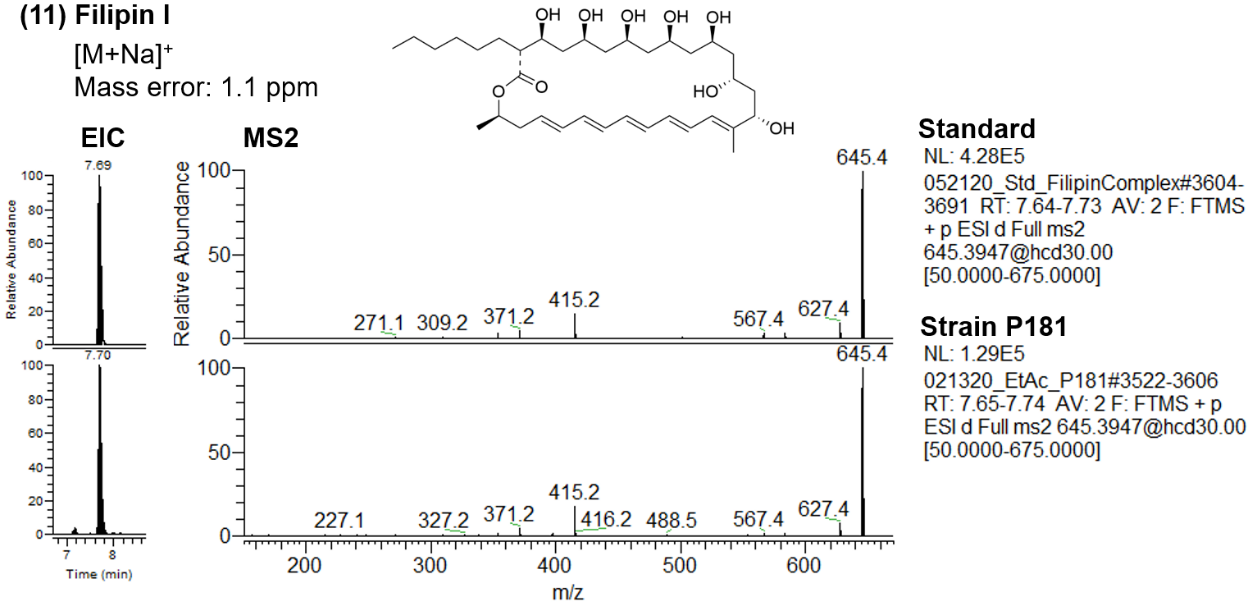

(12) Filipin II

$[M+Na]^+$

Mass error: 1.7 ppm

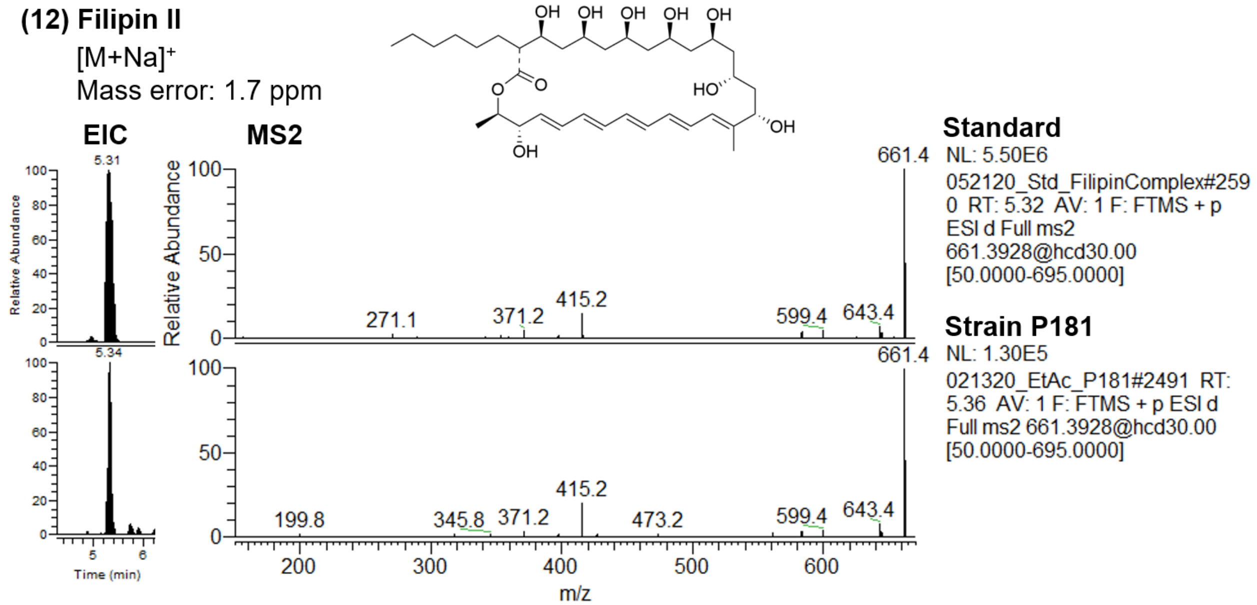

(13) Filipin III

$[M+Na]^+$

Mass error: 0.3 ppm

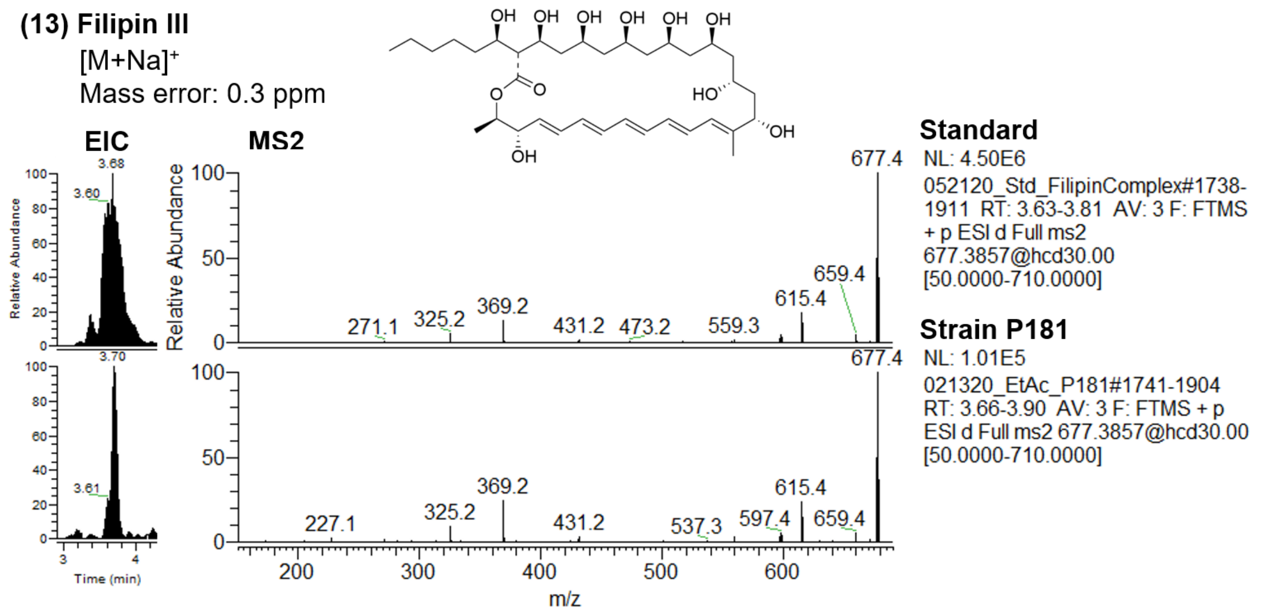

(18) Antimycin A

$[M+H]^+$

Mass error: 0.0 ppm

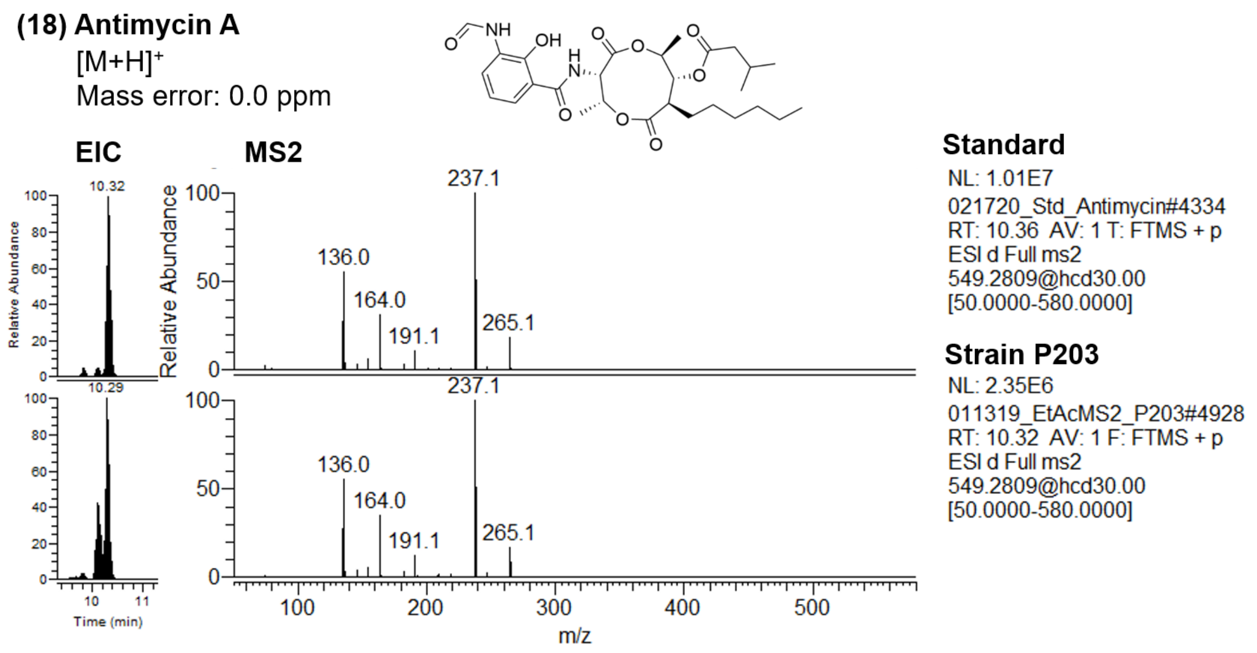

(19) Nocardamine

[M+H]+

Mass error: 0.8 ppm

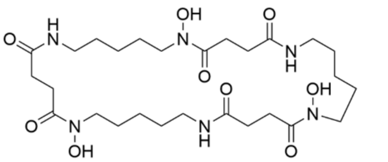

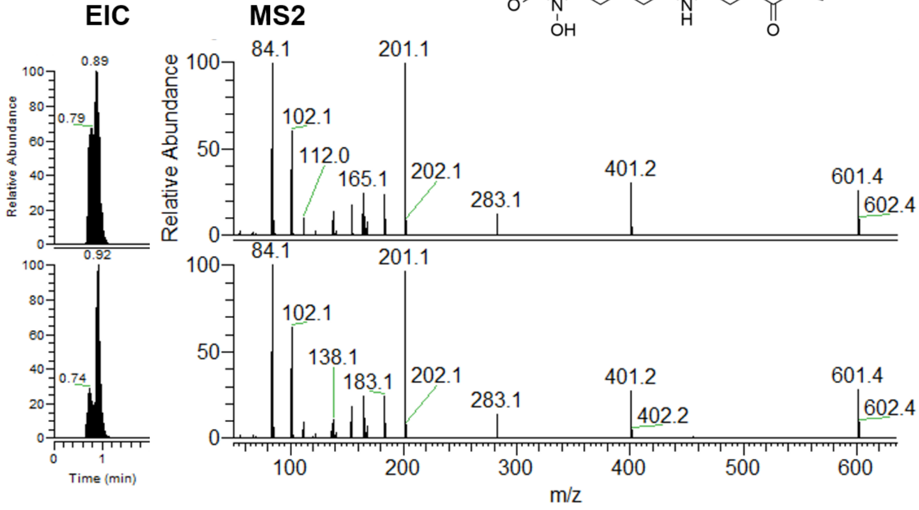

Standard

NL: 9.53E5

022520_Std_Nocardamine#207-442 RT: 0.70-0.96 AV: 4 F: FTMS + p ESI d Full ms2 601.3552@hcd30.00 [50.0000-630.0000]

Strain P307

NL: 9.62E5

011319_EtAcMS2_P307#207-465 RT: 0.70-0.98 AV: 4 F: FTMS + p ESI d Full ms2 601.3552@hcd30.00 [50.0000-630.0000]

(20) Bafilomycin A1

[M+Na]+

Mass error: 1.7 ppm

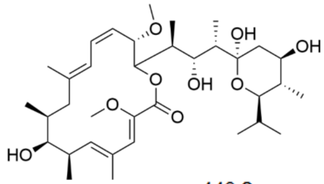

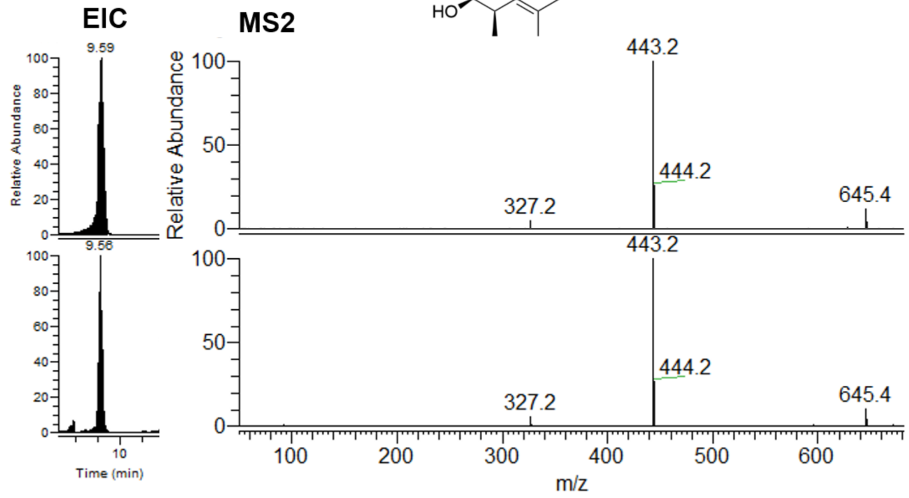

Standard

NL: 6.33E6

100219_BafilomycinA1#3952-4066 RT: 9.54-9.63 AV: 2 F: FTMS + c ESI d Full ms2 645.3981@hcd30.00 [50.0000-675.0000]

Strain P059

NL: 5.72E5

021320_EtAc_P059#4397-4507 RT: 9.51-9.60 AV: 2 F: FTMS + p ESI d Full ms2 645.3964@hcd30.00 [50.0000-675.0000]

(21) Bafilomycin B1

$[M+Na]^+$

Mass error: 2.0 ppm

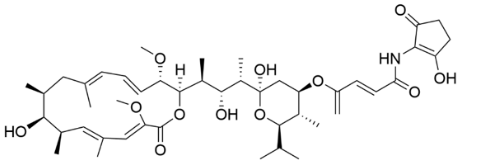

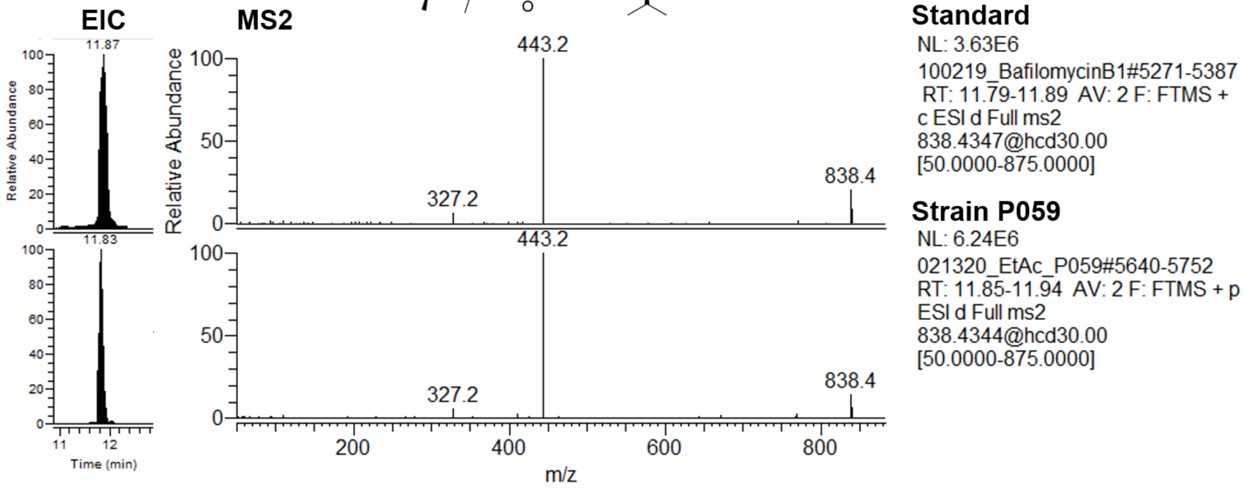

(22) Novobiocin

$[M+H]^+$

Mass error: 2.4 ppm

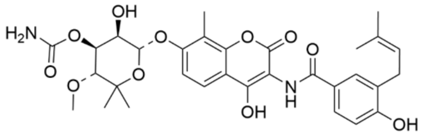

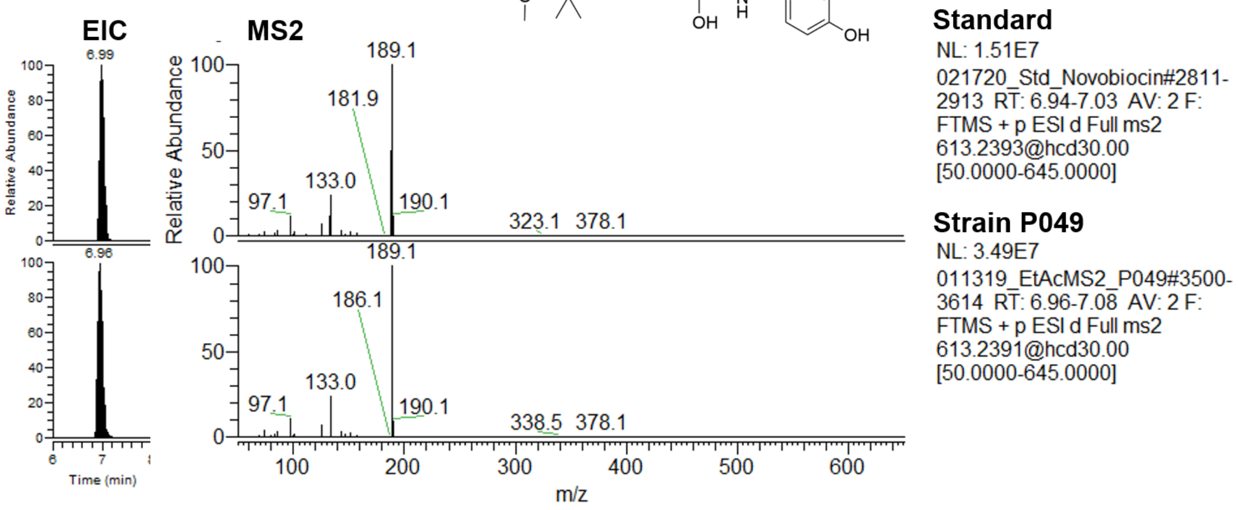

(24) Piericidin A

$[M+H]^+$

Mass error: 2.6 ppm

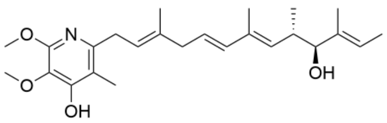

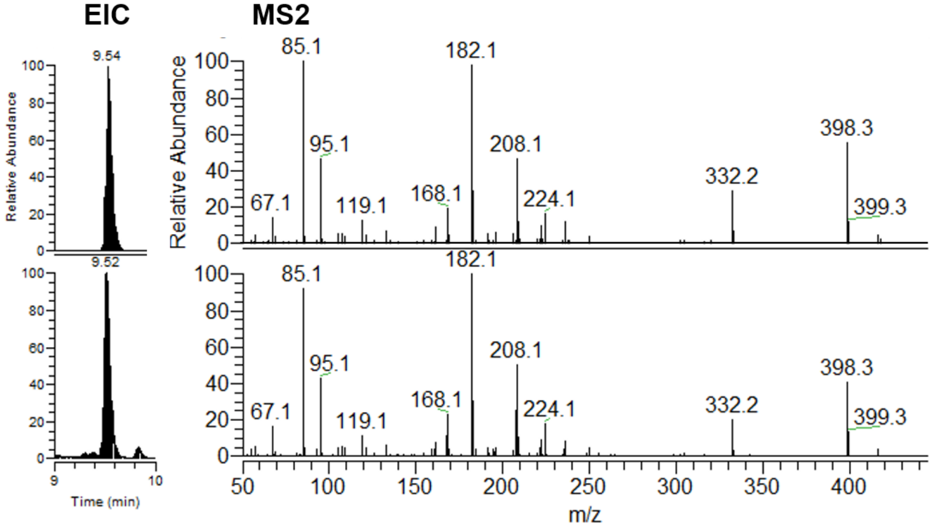

Standard

NL: 2.04E7

100219_Piericidin#4783 RT: 9.53 AV: 1 F: FTMS + c ESI d Full ms2 416.2798@hcd30.00 [50.0000-445.0000]

Strain P190

NL: 2.29E6

021420_EtAc_P190#4166 RT: 9.51 AV: 1 T: FTMS + p ESI d Full ms2 416.2788@hcd30.00 [50.0000-445.0000]

(25) Nigericin

$[M+Na]^+$

Mass error: 0.5 ppm

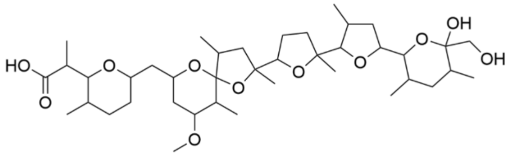

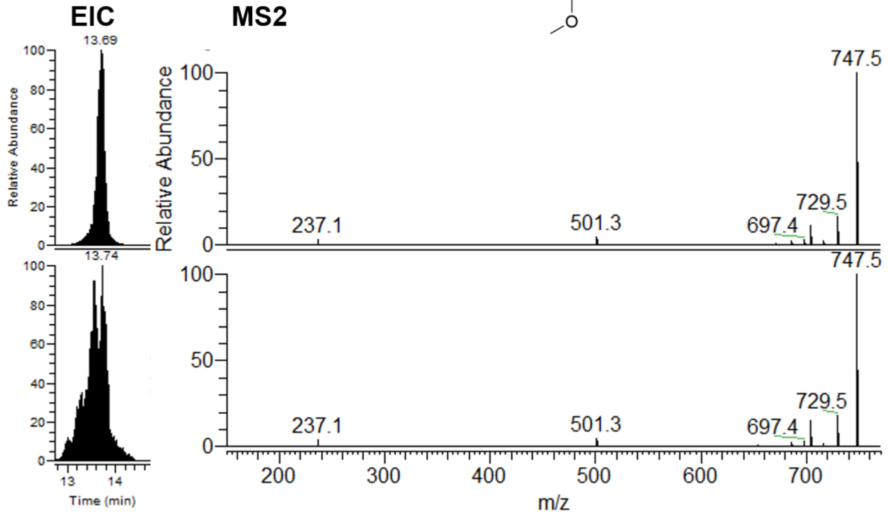

Standard

NL: 1.28E8

052120_Std_Nigericin#5740-5841 RT: 13.64-13.72 AV: 2 F: FTMS + p ESI d Full ms2 747.4639@hcd30.00 [50.0000-780.0000]

Strain P009

NL: 1.98E8

011319_EtAcMS2_P009#7502-7611 RT: 13.61-13.79 AV: 3 F: FTMS + p ESI d Full ms2 747.4639@hcd30.00 [50.0000-780.0000]

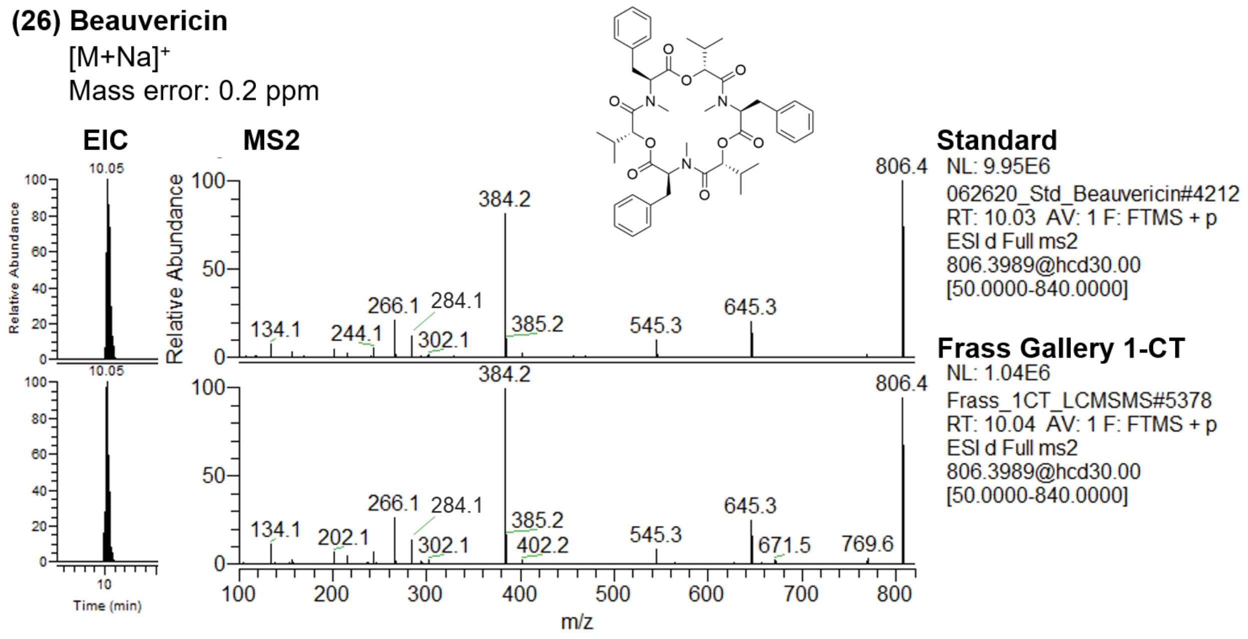

Supplementary File 2: Extracted ion chromatogram (EIC, showing retention time) and MS2 spectra of each compound annotated at identification level 1 (Actinomycin D, Actinomycin X2, STA-21, Rubiginone B2, Cycloheximide, Nonactin, Monactin, Dinactin, Trinactin, Tetranactin, Filipin I, Filipin II, Filipin III, Antimycin A, Nocardamine, Bafilomycin A1, Bafilomycin B1, Novobiocin, Piericidin A, Nigericin, Beauvericin), comparing a commercial standard (top) to the culture extract of an exemplary microbe or environmental frass extract (bottom).
