# Supplementary material for: Multiple lineages of Streptomyces produce antimicrobials within passalid beetle galleries across eastern North America
Source: eLife. 2021 May 4;10:e65091. doi: 10.7554/eLife.65091 (PMC8096431; doi:10.7554/eLife.65091)
Supplement: Supplementary file 3. — Publicly available spectra can be found at: alteramide A, alteramide B: f.MSV000079516/ccms_peak/Labelled/R5_lab_J1074_pre.mzXML; surugamide A: f.MSV000079519/ ccms_peak/Unlabelled/A1_unlab_J1074_pre.mzXML (accessed on June 2020). [file elife-65091-supp3.pdf]

(16) Alteramide A

$[M+H]^+$

Mass error: 2.2 ppm

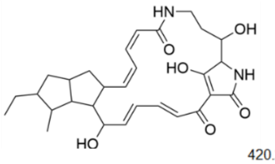

Strain P327, MS2 spectrum of *m/z* 511.2786

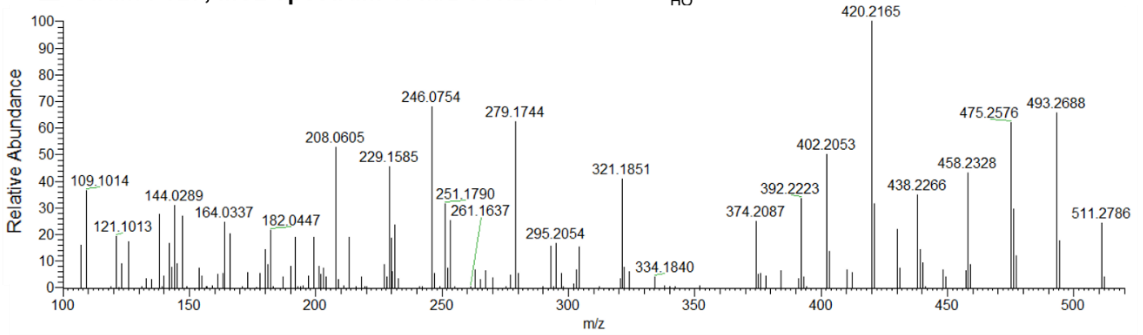

***Streptomyces albus* J1074, MS2 spectrum of *m/z* 511.2800**

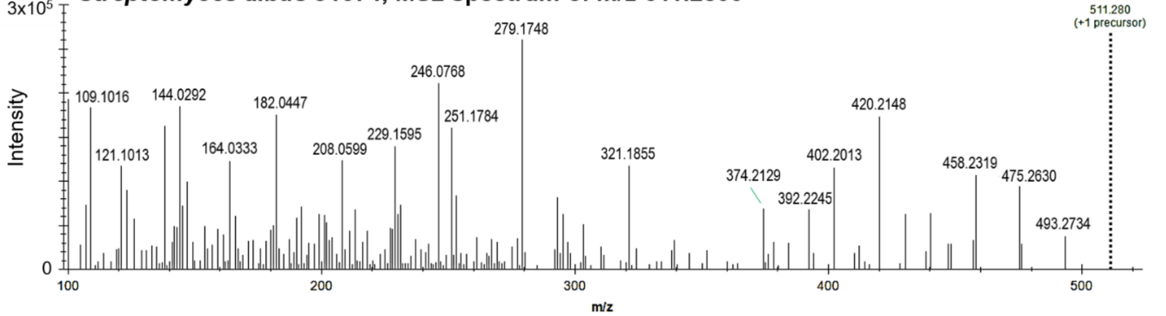

(17) Alteramide B

$[M+H]^+$

Mass error: 4.0 ppm

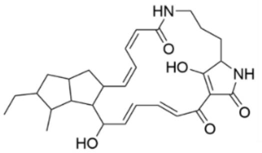

Strain P327, MS2 spectrum of *m/z* 495.2856

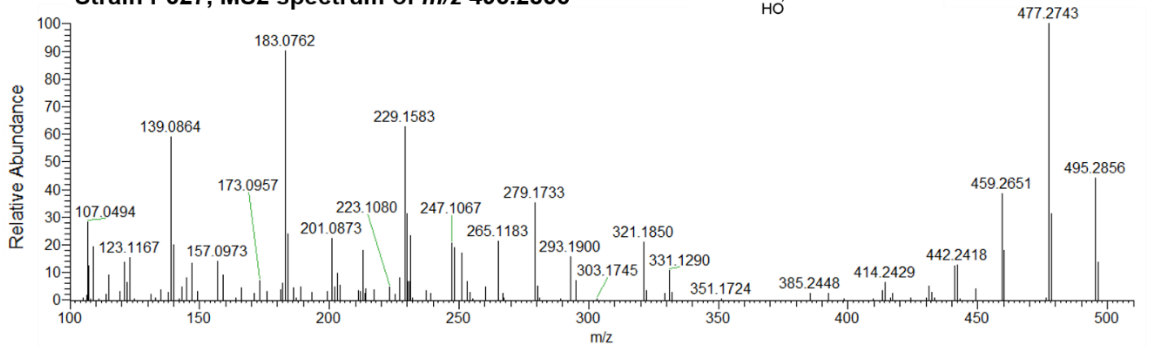

***Streptomyces albus* J1074, MS2 spectrum of *m/z* 495.2848**

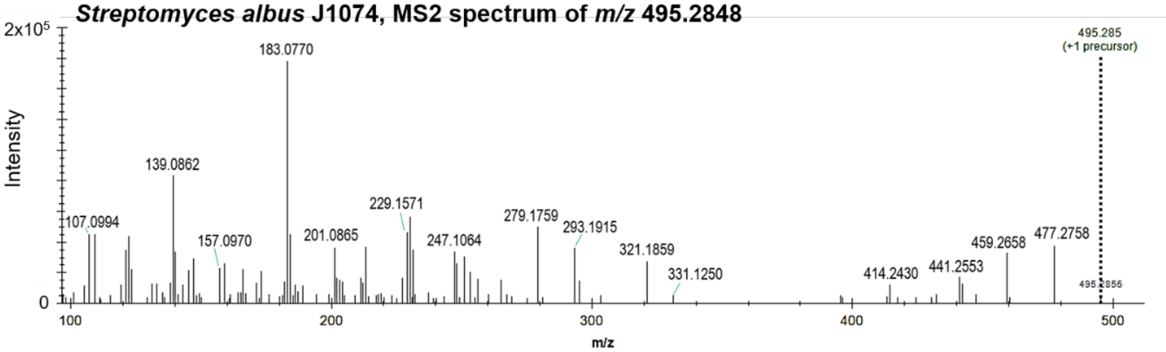

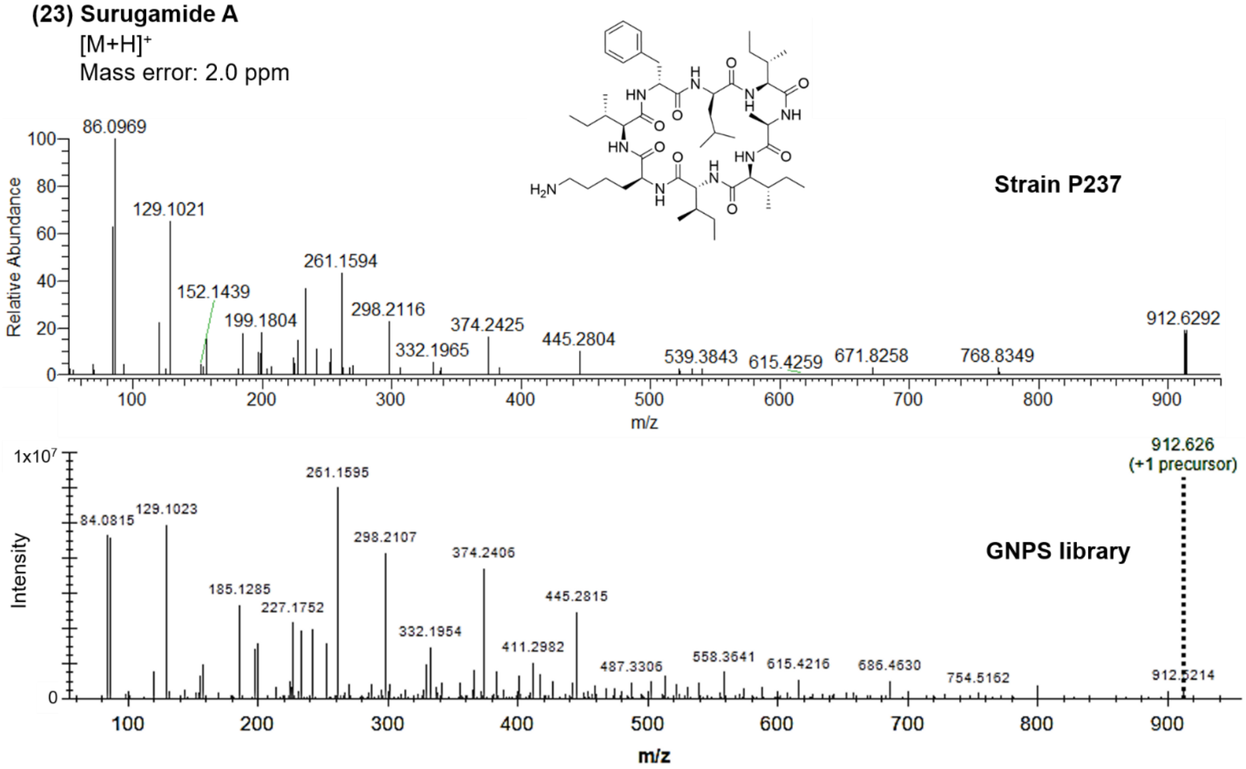

Supplementary File 3: MS2 spectra of each compound annotated at identification level 2 (Alteramide A, Alteramide B, Surugamide A), comparing a spectrum detected in the culture extract of an exemplary microbe (top) to a publicly available spectrum on the MassIVE repository (bottom). Publicly available spectra can be found at: Alteramide A, Alteramide B: f.MSV000079516/ccms_peak/Labelled/R5_lab_J1074_pre.mzXML; Surugamide A: f.MSV000079519/ccms_peak/Unlabelled/A1_unlab_J1074_pre.mzXML (accessed on June/2020).
