# Supplementary material for: Multiple lineages of Streptomyces produce antimicrobials within passalid beetle galleries across eastern North America
Source: eLife. 2021 May 4;10:e65091. doi: 10.7554/eLife.65091 (PMC8096431; doi:10.7554/eLife.65091)
Supplement: Supplementary file 4. — Annotations were made based on fragmentation similarities with other analogs of the same family annotated at identification level 1 (filipins I–III). [file elife-65091-supp4.pdf]

(14) Filipin IV

$[M+Na]^+$

Mass error: 1.0 ppm

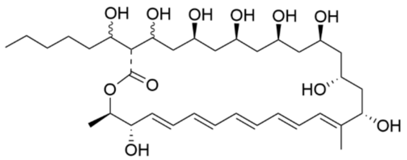

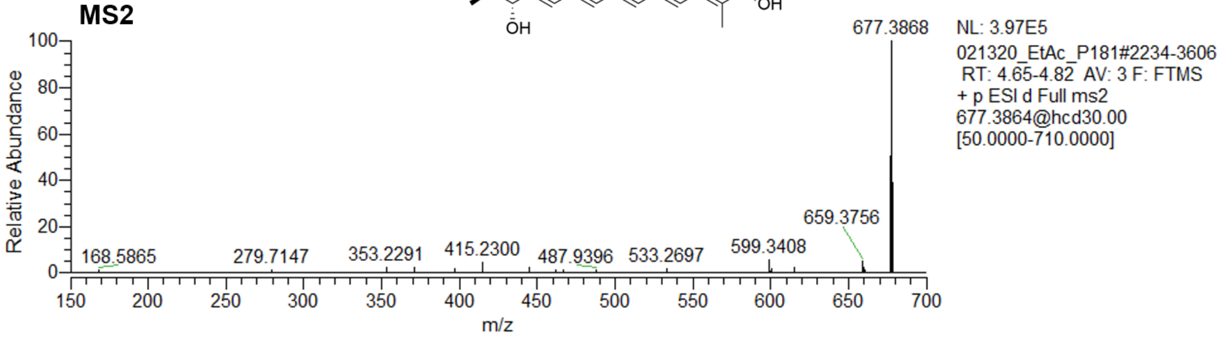

(15) Fungichromin

$[M+Na]^+$

Mass error: 2.2 ppm

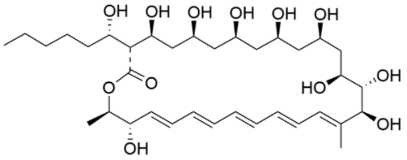

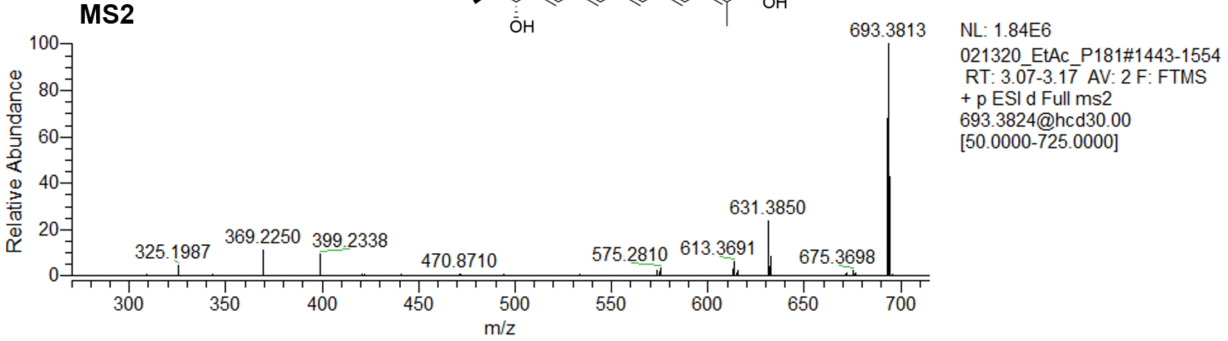

Supplementary File 4: MS2 spectrum of each compound putatively annotated at identification level 3 (Filipin IV, Fungichromin), detected in the culture extract of an exemplary microbe. Annotations were made based on fragmentation similarities with other analogs of the same family annotated at identification level 1 (filipins I-III).
