# Supplementary material for: Multiple lineages of Streptomyces produce antimicrobials within passalid beetle galleries across eastern North America
Source: eLife. 2021 May 4;10:e65091. doi: 10.7554/eLife.65091 (PMC8096431; doi:10.7554/eLife.65091)
Supplement: Supplementary file 5. — (A) Pupal chamber material; (B, C) old frass, plus the extracted ion chromatogram (EIC) of an exemplary compound detected in each sample. The MS1 spectrum refers to the main peak detected on each EIC, highlighting two adducts of each compound. [file elife-65091-supp5.pdf]

**A) Pupal chamber material (gallery 17-LA)**

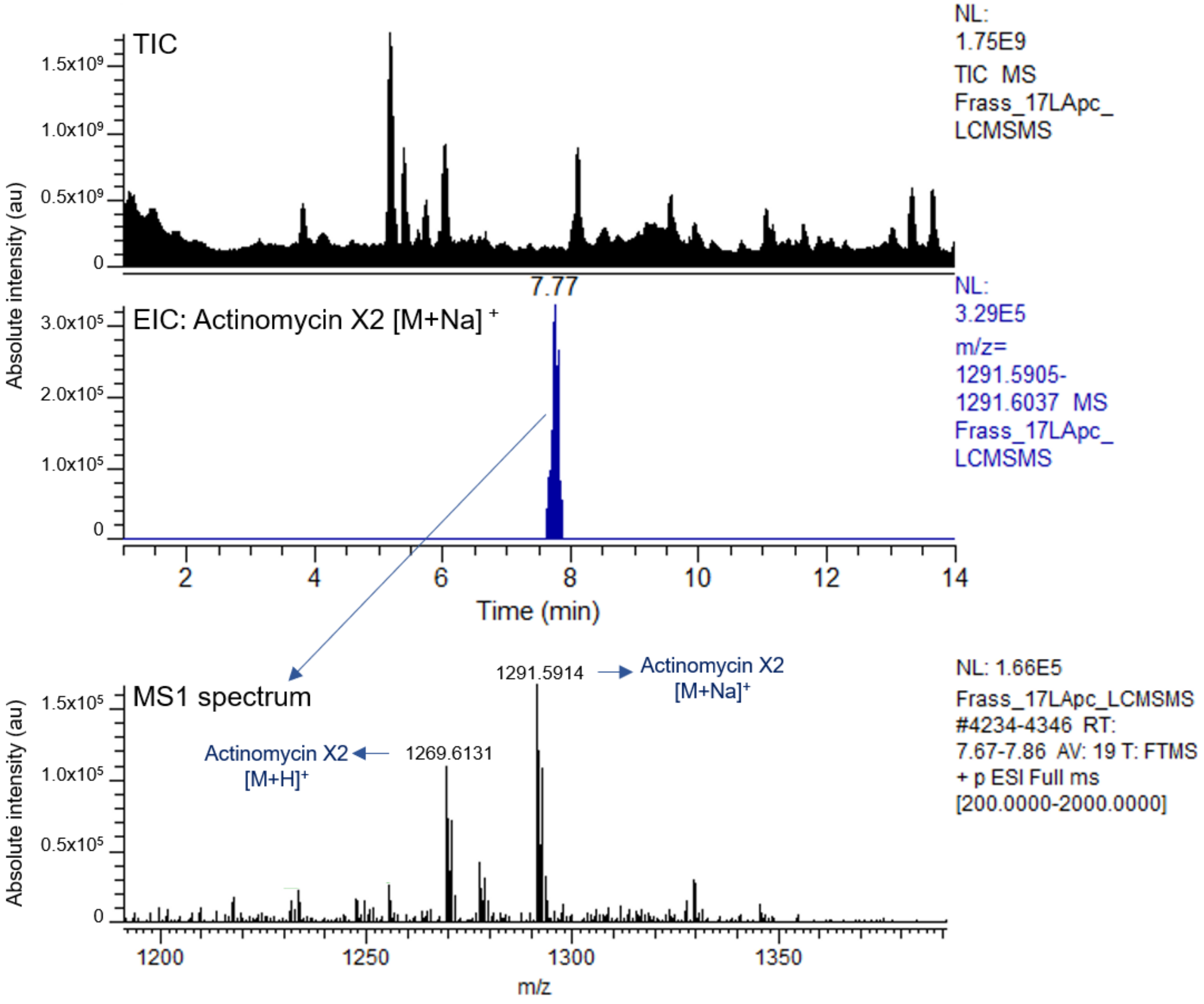

**B) Old frass (gallery 3-DC)**

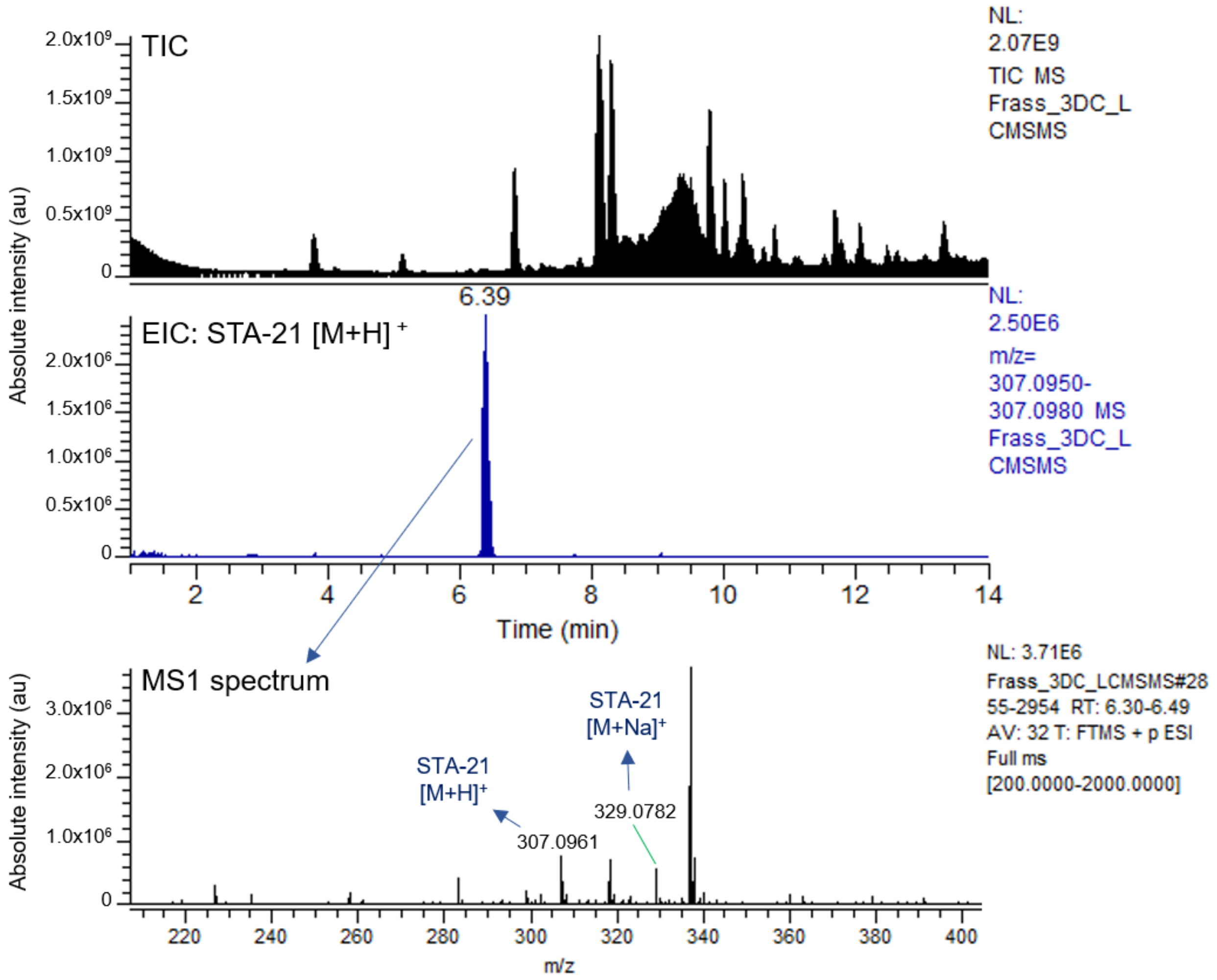

**C) Old frass (gallery 20-LA)**

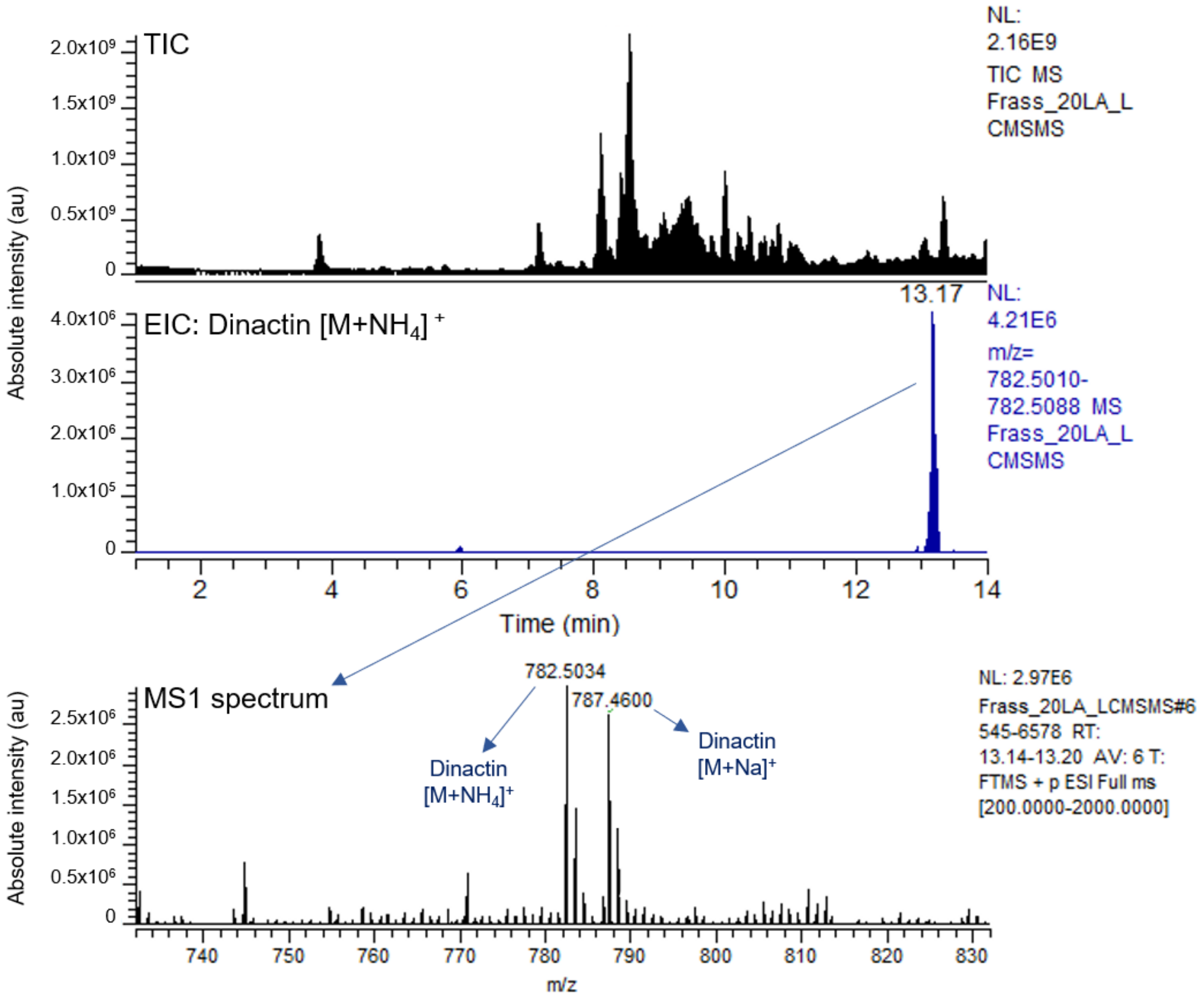


**Supplementary File 5:** Three exemplary total ion chromatograms (TIC) of the LC-MS/MS analysis performed on environmental samples (**A**: pupal chamber material; **B, C:** old frass), plus the extracted ion chromatogram (EIC) of an exemplary compound detected in each sample. The MS1 spectrum refers to the main peak detected on each EIC, highlighting two adducts of each compound.
