# Supplementary material for: Multiple lineages of Streptomyces produce antimicrobials within passalid beetle galleries across eastern North America
Source: eLife. 2021 May 4;10:e65091. doi: 10.7554/eLife.65091 (PMC8096431; doi:10.7554/eLife.65091)
Supplement: Supplementary file 6. — (A) S. padanus P333 growing on an ISP2-agar plate after 7 days of incubation at 30°C. (B) Galleries that S. padanus was isolated from. [file elife-65091-supp6.pdf]

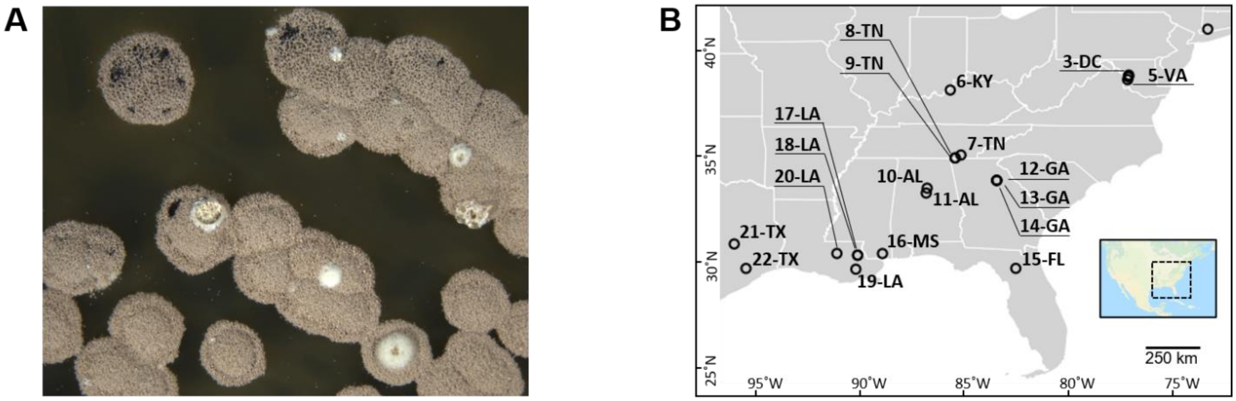

Supplementary File 6: Strains of *Streptomyces padanus* were isolated from 19/22 of the sampled *O. disjunctus* galleries**. A)** *S. padanus* P333 growing on an ISP2-agar plate after seven days of incubation at 30°C. **B)** Galleries that *S. padanus* was isolated from.
