# Supplementary material for: Multiple lineages of Streptomyces produce antimicrobials within passalid beetle galleries across eastern North America
Source: eLife. 2021 May 4;10:e65091. doi: 10.7554/eLife.65091 (PMC8096431; doi:10.7554/eLife.65091)
Supplement: Supplementary file 7. — The figure shows the prevalence of the Streptomyces-derived genes in all four gut compartments with significantly higher normalized-coverage in the posterior hindgut (PHG), the region where frass is compacted prior to its excretion. In each boxplot, a point represents a single gene per category and its detected coverage, and the diamond symbols represent the mean. The box boundaries represent the first and third quartiles of the distribution, and the median is represented as the horizontal line inside each box. Boxplots whiskers span 1.5 times the interquartile range of the distribution. FG: foregut; MG: midgut; AHG: anterior hindgut. Statistical differences were evaluated with Kruskal–Wallis test, and pairwise comparisons were done using a two-sided Wilcoxon test with p-values adjusted using the Benjamini–Hochberg method. Contigs and RPKM-normalized coverage data reported in Ceja-Navarro et al., 2019 were used to generate this figure. Contigs were aligned against the NCBI non-redundant database using the DIAMOND software (Buchfink et al., 2015) and the ‘long reads’ option. The obtained alignment was imported into MEGAN (Huson et al., 2018) and the taxonomy assigned using MEGAN’s LCA algorithm for long reads. Coverage data across the four regions of O. disjunctus’ gut was retrieved for contigs identified as taxonomically derived from Streptomyces sp. [file elife-65091-supp7.pdf]

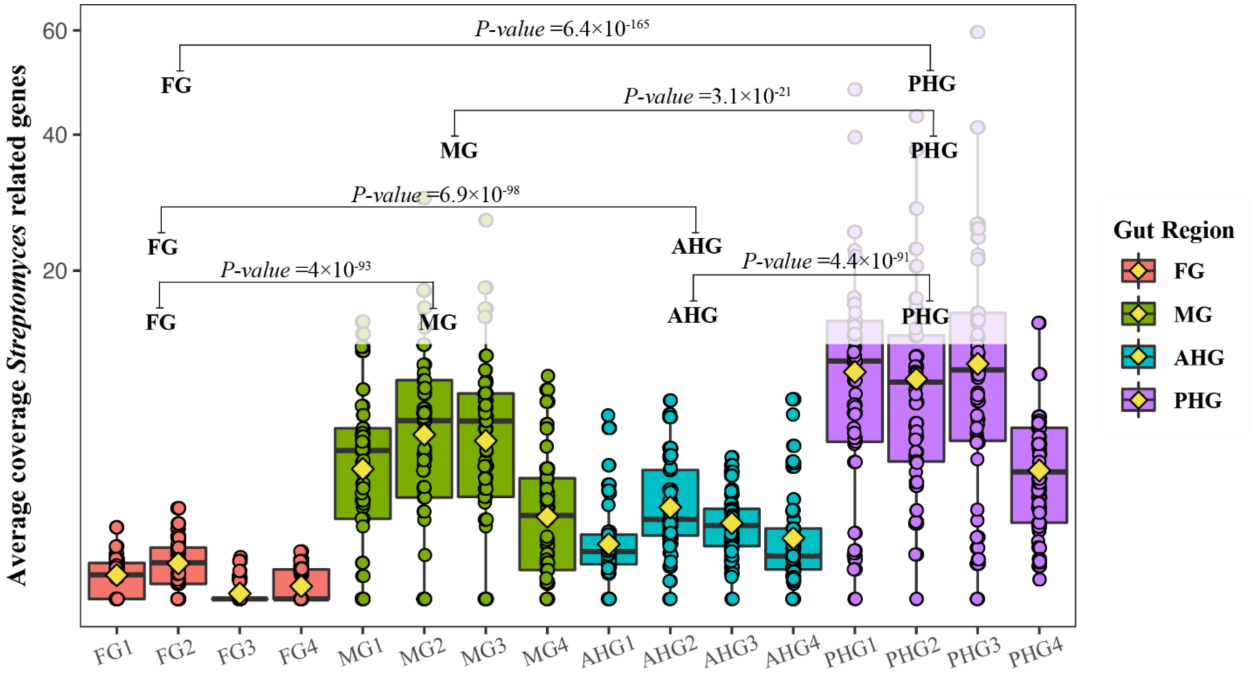

Supplementary File 7: Average coverage distribution of *Streptomyces*-derived genes identified in the metagenome of the different gut compartments of *O. disjunctus*. The figure shows the prevalence of the *Streptomyces*-derived genes in all four gut compartments with significantly higher normalized-coverage in the posterior hindgut (PHG), the region where frass is compacted prior to its excretion. In each boxplot, a point represents a single gene per category and its detected coverage, and the diamond symbols represent the mean. The box boundaries represent the first and third quartiles of the distribution and the median is represented as the horizontal line inside each box. Boxplots whiskers span 1.5 times the interquartile range of the distribution. FG = Foregut, MG = Midgut, AHG = Anterior Hindgut. Statistical differences were evaluated with Kruskal-Wallis test and pairwise comparisons were done using a two-sided Wilcox test with P-values adjusted using the Benjamini-Hochberg method. Contigs and RPKM-normalized coverage data reported in Ceja-Navarro et al. (2019) (Ceja-Navarro et al., 2019) were used to generate this figure. Contigs were aligned against the NCBI non-redundant database using the DIAMOND software (Buchfink et al., 2014) and the "long reads" option. The obtained alignment was imported into MEGAN (Huson et al., 2018) and the taxonomy assigned using MEGAN's LCA algorithm for long reads. Coverage data across the four regions of *O. disjunctus*' gut was retrieved for contigs identified as taxonomically-derived from *Streptomyces* sp.
