# Supplementary material for: Multiple lineages of Streptomyces produce antimicrobials within passalid beetle galleries across eastern North America
Source: eLife. 2021 May 4;10:e65091. doi: 10.7554/eLife.65091 (PMC8096431; doi:10.7554/eLife.65091)
Supplement: Supplementary file 8. — Magnification: 7×. [file elife-65091-supp8.pdf]

Metarhizium anisopliae P016 *Metarhizium anisopliae* P287

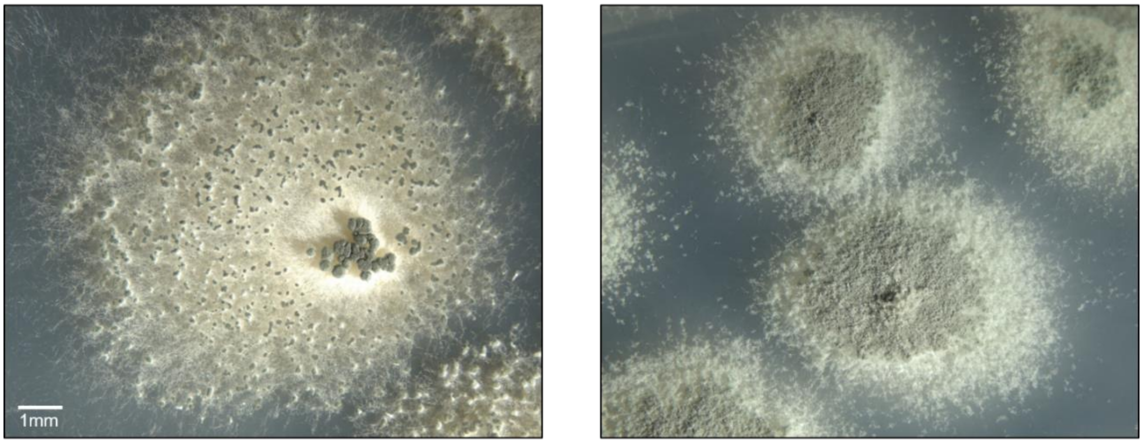

Supplementary File 8: *Metarhizium anisopliae* strains P016 and P287 phenotypes after 10 days growing on PDA plates incubated at 25°C under constant light. Magnification: 7x.
